# Outcome of 2 cc pure sulfur hexafluoride gas tamponade for macular hole surgery

**DOI:** 10.1186/s12886-016-0254-9

**Published:** 2016-06-03

**Authors:** Naresh B. Kannan, Olukorede O. Adenuga, Karthik Kumar, Kim Ramasamy

**Affiliations:** Aravind Eye Hospitals and Postgraduate Institute of Ophthalmology, 1 Anna Nagar, Madurai, 625 020 Tamil Nadu India; Jos University Teaching Hospital, Jos, Plateau State Nigeria

**Keywords:** Macular hole surgery, Vitrectomy, Sulfur hexafluoride

## Abstract

**Background:**

Isoexpansile concentrations of intraocular gases are typically used as tamponading agent in macular hole surgery. Using a small volume of the pure form of these gases may achieve the same result without increasing the incidence of postoperative complications. The purpose of this study was to evaluate the anatomical and visual outcomes following macular hole surgery with 2 cc pure (100 %) sulfur hexafluoride (SF_6_) gas tamponade.

**Methods:**

A retrospective study of eyes with idiopathic macular holes that underwent 23-gauge pars plana vitrectomy with 2 cc pure SF_6_ gas tamponade. Macular hole surgery was performed alone or in combination with phacoemulsification in eyes with cataract. Preoperative and postoperative data including best corrected visual acuity recorded in LogMAR units, slit-lamp biomicroscopy, and optical coherence tomography were analysed. Surgical complications were also recorded.

**Results:**

Seventy six eyes of seventy five patients were analysed. A closure rate of 100 % was achieved with reoperation in 4 eyes. There was a significant improvement in best-corrected visual acuity from a mean of 0.65 LogMAR preoperatively to 0.36 at 6 months (*p* value 0.004). Forty five (59 %) eyes gained at least 2 lines on the Snellen visual acuity chart. Postoperative elevation in intraocular pressure (≥30 mmHg) was documented in 3 eyes (4 %).

**Conclusion:**

Macular hole surgery with 2 cc pure SF_6_ gas tamponade achieved a high success rate with a low incidence of complications. The smaller volume of gas required makes it a cheaper technique.

**Electronic supplementary material:**

The online version of this article (doi:10.1186/s12886-016-0254-9) contains supplementary material, which is available to authorized users.

## Background

Recent advances in surgical techniques and instrumentation have made Macular Hole Surgery (MHS) now one of the most successful and gratifying procedures for vitreoretinal surgeons [1]. The first report of MHS was published in 1991 by Kelly and Wendel who achieved a 58 % closure rate in 52 operated eyes [2]. Today, however, success rates of over 90 % have been recorded [3–7]. Macular hole surgery typically involves Pars Plana Vitrectomy (PPV), Internal Limiting Membrane (ILM) peeling and internal gas tamponade [8]. The commonly used gases are Sulfur Hexafluoride (SF_6_) and Perfluoropropane (C_3_F_8_) with the shorter acting SF_6_ being preferred by Japanese and American retinal surgeons [9, 10]. Sulfur hexafluoride achieves similar results to C_3_F_8_ and is absorbed faster, allowing for quicker visual rehabilitation [6].

When used to fill the posterior segment during surgery, intraocular gases are usually diluted with air because of the tendency of the pure forms of these gases to expand within the eye [9]. A recent survey of the American Society of Retina Specialists (ASRS) revealed that 90 % of respondents used a full gas fill with an isoexpansile mixture, 9 % used injection of pure gas into an estimated eye volume to achieve a desired concentration, and 1 % manually diluted pure gas in a large syringe during air-gas exchange. When using pure SF_6_ or C_3_F_8_ a volume must be chosen so that subsequent expansion of the gas does not result in a dangerous elevation of Intraocular Pressure (IOP) [11]. The aim of this study was to assess the anatomical and visual outcomes with 2 cc pure SF_6_ gas tamponade for MHS.

## Methods

This was a retrospective case series involving eyes with macular holes that underwent MHS with 2 cc pure (100 %) SF_6_ gas tamponade at Aravind Eye Hospital in Madurai, India. The study adhered to the tenets of the declaration of Helsinki and was approved by the Institutional Review Board of the hospital (IRB number: 20122016CLI). Surgeries were carried out over a period of 32 months from July 2011 to February 2014. Inclusion criteria included eyes with idiopathic stage 2–4 macular holes and postoperative follow-up of at least 6 months duration. Exclusion criteria were eyes with traumatic macular holes, high myopia (≥ minus 6D), previous PPV and co-existing ocular co-morbidity such as retinal detachment, glaucoma or diabetic retinopathy.

Demographic data including age and gender were obtained for each patient as well as duration of symptoms and involved eye. Preoperative evaluation included Best-Corrected Visual Acuity (BCVA) assessment with the Snellen Visual Acuity (VA) chart, slit lamp examination of the anterior segment, IOP measurement and a dilated fundus examination. A macular hole was diagnosed by slit lamp biomicroscopy with a 90D lens and confirmed by Spectral Domain Optical Coherence Tomography (Spectralis HRA + OCT by Heidelberg Engineering, Heidelberg, Germany). The basal diameter of the macular hole was measured on OCT in all cases.

Macular hole surgery was performed with or without phacoemulsification. A written informed consent was obtained from each patient prior to surgery. Combination with phacoemulsification was done if a cataract was present. Surgeries were done under peribulbar local anaesthesia. Phacoemulsification with implantation of an acrylic foldable Intraocular Lens (IOL) in the capsular bag was done by a cataract surgeon just before the MHS in cases of combined surgery. Cataract surgeries were performed by more than one surgeon while all the macular hole surgeries were carried out by a single experienced surgeon. A PPV with 23-gauge, standard 3-port approach was done in each case. Central core vitrectomy was performed followed by detachment of the posterior hyaloid (for stage 2 and 3 macular holes) using high vacuum of the vitrectomy probe and assisted by intravitreal triamcinolone injection. The peripheral vitreous was then removed with careful inspection of the retinal periphery. The macular area was stained with brilliant blue G dye 0.05 % (Ocublue plus by Aurolab, India) and peeling of the ILM carried out. Where an epiretinal membrane was present it was removed before peeling the ILM. A complete fluid-air exchange was performed and the superonasal and superotemporal cannulae removed and the conjunctiva repositioned to cover the sclerotomy sites. Injection of 2 cc pure SF_6_ with a 30-gauge needle inserted through the pars plana superiorly was then done with the air-infusion line used for venting. After the syringe was flushed, the infusion line was clamped and the digital tension of the globe assessed. The infusion cannula was then removed and the inferotemporal sclerotomy sealed. Patients were advised to maintain a face-down position for 7 days.

Postoperatively topical steroids, antibiotics and cycloplegics were prescribed and gradually tapered. Patients were examined on day 1, at 2 weeks, 6 weeks, 3 months and then at 6 months postoperatively. Gas fill of the eye and IOP were assessed on the first postoperative day and at 2 weeks. Best-corrected visual acuity and IOP were measured at each visit and a slit lamp biomicroscopy with a 90D lens done to assess the status of the hole. Postoperative complications were documented when present. All the patients had an OCT done at 2 weeks to confirm the status of the hole.

Data analysis was done using Epi Info 7.1.5.0. Best-corrected visual acuity was recorded as a Snellen visual acuity and converted to LogMAR for statistical analysis. Frequencies and percentages were computed for qualitative variables like gender, stage of macular hole, and preoperative lens status. Mean and standard deviation was computed for quantitative variables such as age, macular hole diameter, BCVA and IOP. Tests for statistical significance were done using Mann-Whitney/Wilcoxon two-sample test (Kruskal-Wallis test for two groups). A *p*-value of less than 0.05 was considered statistically significant. Visual improvement was defined as an increase in BCVA by 2 or more lines on the Snellen VA chart at 6 months follow-up while anatomical success was defined as hole closure on OCT. The primary outcome measures were anatomical success and visual improvement while secondary outcome was postoperative complications. At follow-up, macular holes were categorized as either open or, if closed, as type 1 or type 2 as earlier described by Kang et al [12]. Type 1 closure refers to closure of the macular hole without foveal defect of the neurosensory retina while type 2 closure indicates a persistent foveal defect of the neurosensory retina even though the whole rim of the macular hole is attached to the underlying retinal pigment epithelium with flattening of the cuff.

## Results

A total of 76 consecutive eyes of 75 patients met the study criteria and were analysed. One patient had bilateral surgery. These were made up of 33 males (44 %) and 42 females (56 %) giving a male:female ratio of 1:1.3. The mean age of the patients was 61.8 years (SD 5.6). Sixty three eyes were phakic (83 %) and 13 eyes pseudophakic (17 %). The clinical characteristics of the macular holes and stages are shown in Tables 1 and 2. Forty nine eyes (64.5 %) with varying degrees of immature cataracts had phacoemulsification in combination with MHS. These constituted 78 % of the phakic eyes.

**Table 1:** Macular hole characteristics

	Mean	Range
Duration of macular hole (months)	4.5 ± 2.5	0.3–12
Hole size (μm)	523.68 ± 233	234–1802

**Table 2:** Stage of Macular Hole

Stage	Frequency (%)
2	10 (13)
3	9 (12)
4	57 (75)
Total	76 (100)

**Fig. 1:**
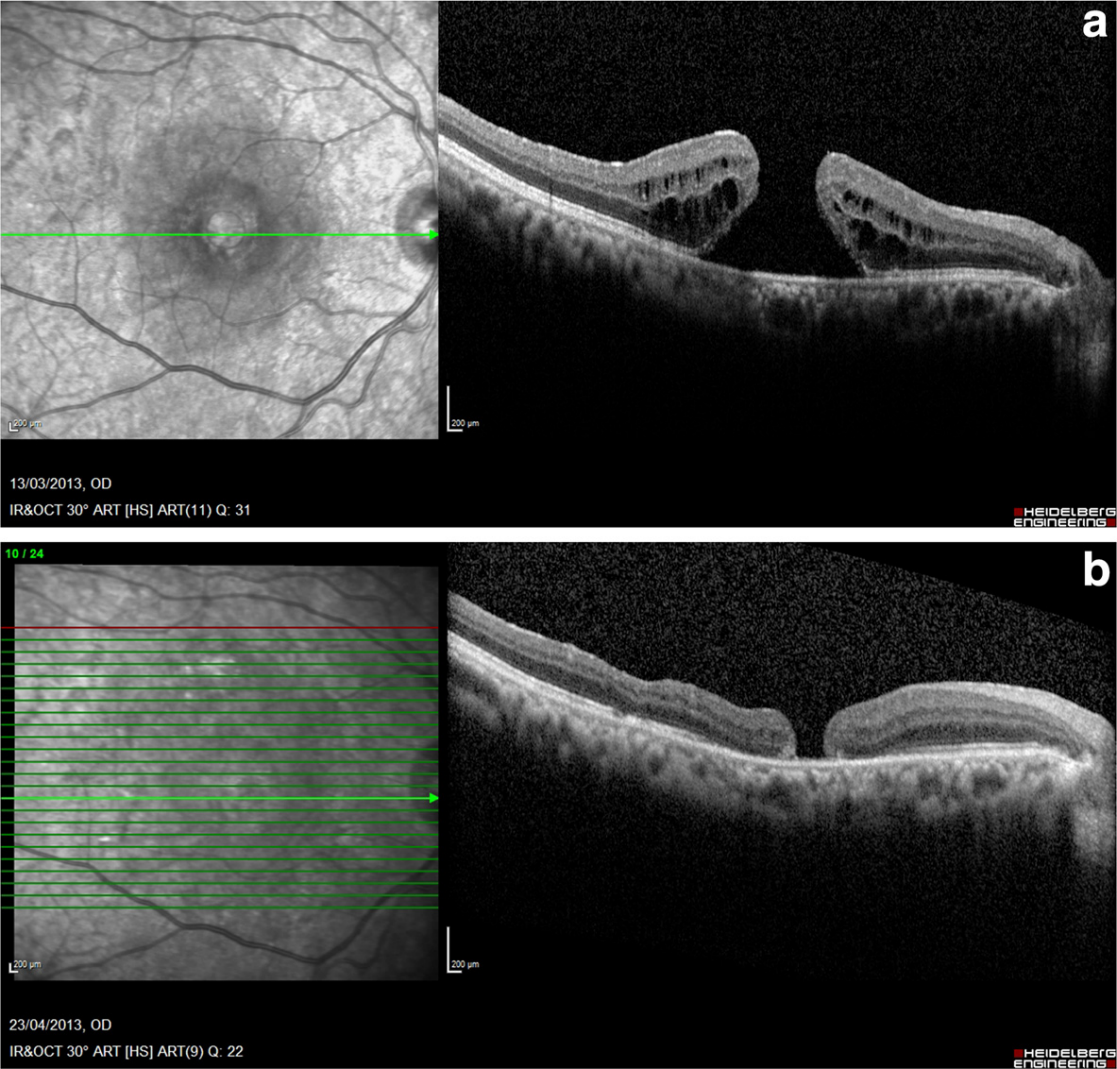
OCT of type 2 macular hole closure. **a** Preoperative OCT. **b** Postoperative OCT

Mean intraocular gas bubble size on the first postoperative day was 73 % (SD 6.1) with a range of 50–90 %. The gas bubble had completely absorbed by the first postoperative visit at 2 weeks in all the eyes. Closure of the hole was achieved at first surgery in 72 eyes (94.7 %). Seventy eyes (92 %) had a type 1 closure while 2 eyes (2.6 %) with large holes had a type 2 closure (Fig. 1). Four eyes in which the hole remained open postoperatively at 2 weeks had a reoperation with fluid-air exchange followed by injection with 2 cc pure SF_6_. The hole was successfully closed in each case. Anatomical success was therefore achieved in 94.7 % of eyes at first surgery and in 100 % with resurgery.

There was a statistically significant improvement in BCVA from a mean of 0.65 LogMAR preoperatively to 0.36 (*p* value 0.004) at 6 months. Sixty eyes (79 %) had an improvement in vision with 45 eyes (59 %) gaining at least 2 lines on the Snellen VA chart. Fourteen eyes (18 %) had no change in BCVA while 2 (2.6 %) eyes lost a line. The mean improvement in Snellen acuity was 1.86 lines (SD 1.36).

There were no intraoperative complications. A mild increase in mean IOP from 14.8 mmHg preoperatively to 16.23 mmHg on the first postoperative day was observed. This difference was statistically significant with a *p* value of 0.01. Three (4 %) eyes had elevated IOP (≥30 mmHg) and were prescribed topical antiglaucoma medications with the IOP returning to normal in 2 eyes at the first follow-up visit at 2 weeks. The third patient had severe postoperative anterior segment inflammation following combined surgery and had to be on treatment for 6 weeks. No case of ocular hypotony was recorded. One patient developed a visually significant cataract 4 months after surgery and underwent phacoemulsification with IOL implantation. No other long term complications were encountered. The raw data for this study is available as Additional file 1.

## Discussion

Since the first report of successful closure of idiopathic macular holes with PPV and fluid-gas exchange in 1991 [2], there have been several modifications made to improve surgical outcomes. Macular hole surgery requires internal gas tamponade and a range of gas types and mixtures have been used with good success [6]. The intraocular gas provides isolation or waterproofing of the macular hole from the vitreous cavity by surface-tension at the gas-liquid interface. It also mechanically tamponades the hole and provides a template over which a nascent bridging membrane forms [13]. In this study, we achieved a closure rate of 100 % with 2 cc pure SF6 gas tamponade. This compares favourably with results obtained with isoexpansile gas mixtures [3–7]. Peeling of the ILM is a likely contributor to this excellent closure rate as it has been shown to significantly improve anatomic success in all stages of chronic and recent macular holes as well as in macula hole retinal detachments [3, 14, 15]. It is believed to facilitate macular hole closure by removing an element of traction or by stimulating gliosis [6].

Intraocular gas volume on the first postoperative day is an important index of postoperative gas dynamics because less gas volume would lead to insufficient tamponade to the retina [16]. A gas bubble size of 73 % obtained on the first postoperative day in this study is lower than 83.8 % reported by Kusuhara et al [16] with 25 % SF_6_. Eyes treated with 23-gauge transconjunctival vitrectomy tend to have earlier gas disappearance or incomplete gas fill as a result of postoperative gas leakage through unsutured sclerotomies. After MHS, however, the most important feature of the gas bubble is its surface tension, which leads to closure of the hole by keeping its edge dry, independent of buoyancy [17]. The surface tension represents contact around the entire interface with the retina [17]. A gas bubble size of 75 % will make an arc of contact with the retina of 240^0^ [18]. Another important factor in gas bubble dynamics which is required to maintain a therapeutic effect after surgery is the interval it takes for the gas to dissipate to a 50 % gas fill (Gas50). This has been used as an index of early postoperative decrease in intraocular gas volume and postoperative gas longevity [16]. Twenty three-gauge PPV which was used in this series is associated with a shorter Gas50 than 20-gauge PPV [16]. Though the Gas50 was not measured in this study, the excellent closure rate suggests that the average gas bubble size of 73 % obtained with 2 cc SF_6_ on the first postoperative day, as well as the duration of the gas bubble were sufficient to accomplish hole closure.

The aim of MHS is to improve the patient’s vision and prevent further visual deterioration [19]. In this present series there was a significant improvement in postoperative vision. This is in agreement with previous studies [3, 4, 6, 17]. The mean postoperative BCVA of LogMAR 0.36 in this series is slightly better than 0.49 obtained by Kim et al [6]. This difference is due to the better preoperative BCVA in this study. Preoperative visual acuity is one of the most important prognostic factors for a good visual outcome following MHS with eyes having a good preoperative visual acuity more likely to attain a better postoperative visual acuity [19, 20]. The proportion of eyes in this study that improved by at least 2 lines on the Snellen chart (59 %) was, however, lower than over 70 % reported by other authors [21, 22].

Several complications may occur with intraocular gas tamponade such as increased IOP, inadequate bubble size, cataract formation, visual field defects, retinal breaks and proliferative vitreoretinopathy [13]. The main concern on day 1, however, is raised IOP and the risk of this occurring has been estimated to be 20–21 % [23]. The increase is usually transient occurring in the majority of eyes within 24 h of surgery and can be managed with topical or systemic anti-glaucoma agents [13, 24]. In a survey on the use of long-lasting expanding gases in ophthalmology in Japan, IOP rise was the most common complication occurring in 15 % of cases involving PPV with the use of expansile gases [25]. The incidence of significant IOP spike with 2 cc SF_6_ in this present study was 4 % which is lower than 13 % reported by Xirou and colleagues [21] using 20-gauge instruments and isoexpansile gas tampomade. Heath and Rahman [26], however, did not record this complication in their series with 23-gauge PPV. Studies have shown that mean postoperative IOP on day 1 as well as the incidence of IOP spike are higher following 20-gauge PPV compared with 23-gauge PPV [15, 27, 28]. Hypotony on the other hand is more likely with sutureless 23-gauge vitrectomy [15, 27, 28]. Hypotony was not encountered in this current series.

One case of visually significant cataract was documented postoperatively in this study. The incidence of this complication was very low as majority of the patients either had combined surgery or had previously undergone cataract extraction. Cataract progression or formation may result from oxidative stress to the lens and trauma following vitrectomy [13]. Prolonged contact of the intraocular gas with the posterior lens surface can also lead to gas-induced cataract, presenting typically as ‘lens feathering’, a manifestation of the branching pattern of posterior subcapsular lens changes [13].

Using 2 cc pure SF_6_ as gas tamponade for MHS has some advantages over the non-expansile concentration of the gas. First is that a smaller volume of the gas is required making this technique cost effective. When using a 20 cc syringe to flush a non-expansile concentration of 20 % SF_6_ gas following fluid-air exchange, 4 cc of SF_6_ will be required and more if a 50 cc syringe is used. Secondly, no cumbersome calculations are required to calculate the volume of gas needed. The chances of errors occurring are, therefore, much less.

The limitations of this study include its retrospective nature, lack of a control group and the relatively small number of cases analysed. Our excellent results, however, provide useful data that could aid in the planning of further prospective, randomized clinical studies such as the outcome of this technique with smaller gauge instruments. The use of smaller volumes of other expansile gases such as C_3_F_8_ in MHS can also be studied.

## Conclusion

In conclusion, 2 cc pure SF_6_ gas tamponade for MHS achieved excellent results with a low incidence of complications. It is a simple technique as no calculations are required to determine the volume of gas to be injected. The smaller volume of gas required compared with using a non-expansile concentration also makes it cost effective.

## Abbreviations

BCVA, Best Corrected Visual Acuity; C_3_F_8_, Perfluoropropane; IOP, Intraocular Pressure; LogMAR, Minimum Angle of Resolution; MHS, Macular Hole Surgery; OCT, Optical Coherence Tomography; PPV, Pars Plana Vitrectomy; SF_6_, Sulfur Hexafluoride; VA, Visual Acuity.

## Additional file

Additional file 1: Raw data. (XLSX 19 kb)

## References

[1] Yuan A (2013). Update on surgery for macular hole and macular pucker. Retin Physician.

[2] Kelly NE, Wendel RT (1991). Vitreous surgery for idiopathic macular holes. Results of a pilot study. Arch Ophthalmol.

[3] Brooks HL (2000). Macular hole surgery with and without internal limiting membrane peeling. Ophthalmology.

[4] Guillaubey A, Malvitte L, Lafontaine PO, Jay N, Hubert I, Bron A (2008). Comparison of face-down and seated position after idiopathic macular hole surgery: a randomized clinical trial. Am J Ophthalmol.

[5] Passemard M, Yakoubi Y, Muselier A, Hubert I, Guillaubey A, Bron AM (2010). Long-term outcome of idiopathic macular hole surgery. Am J Ophthalmol.

[6] Kim SS, Smiddy WE, Feuer WJ, Shi W (2008). Outcomes of sulfur hexafluoride (SF6) versus perfluoropropane (C3F8) gas tamponade for macular hole surgery. Retina.

[7] Tadayoni R, Vicaut E, Devin F, Creuzot-Garcher C, Berrod JP, Le Mer Y (2011). A randomized controlled trial of alleviated positioning after small macular hole surgery. Ophthalmology.

[8] Kanski JJ, Bowling B (2011). Clinical ophthalmology: a systemic approach.

[9] Sigler EJ, Randolph JC, Charles S, Jorge I, Calzada JI (2012). Intravitreal fluorinated gas preference and occurrence of rare ischemic postoperative complications after pars plana vitrectomy: a survey of the American Society of Retina Specialists. J Ophthalmol.

[10] Donati S, Caprani SM, Airaghi G, Vinciguerra R, Bartalena L, Testa F (2014). Vitreous substitutes: the present and the future. BioMed Res Int.

[11] Jacobs PM, Twomey JM, Leaver PK (1988). Behaviour of intraocular gases. Eye.

[12] Kang SW, Ahn K, Ham D-I (2003). Types of macular hole closure and their clinical implications. Br J Ophthalmol.

[13] Mohamed S, Lai TYY (2010). Intraocular gas in vitreoretinal surgery. Hong Kong J Ophthalmol.

[14] Christensen UC, Kroyer K, Sander B, Larsen M, Henning V, Villumsen J (2009). Value of internal limiting membrane peeling in surgery for idiopathic macular hole stage 2 and 3: a randomised clinical trial. Br J Ophthalmol.

[15] Mancino R, Ciuffoletti E, Martucci A, Aiello F, Cedrone C, Cerulli L (2013). Anatomical and functional results of macular hole retinal detachment surgery in patients with high myopia and posterior staphyloma treated with perfluoropropane gas or silicone oil. Retina.

[16] Kusuhara S, Ooto S, Kimura D, Itoi K, Mukuno H, Miyamoto N (2011). Intraocular gas dynamics after 20-gauge and 23-gauge vitrectomy with sulfur hexafluoride gas tamponade. Retina.

[17] Yagi F, Takagi S, Tomita G (2012). Combined idiopathic macular hole vitrectomy with phacoemulsification without face-down positioning. J Ophthalmol.

[18] Martinez-Toldos JJ, Hovos JE (2013). Step by step: vitrectomy.

[19] Jaycock PD, Bunce C, Xing W, Thomas D, Poon W, Gazzard G (2005). Outcomes of macular hole surgery: implications for surgical management and clinical governance. Eye.

[20] Kusuhara S, Negi A (2014). Predicting visual outcome following surgery for idiopathic macular holes. Ophthalmologica.

[21] Xirou T, Theodossiadis PG, Apostolopoulos M, Kabanarou SA, Feretis E, Ladas ID (2012). Macular hole surgery with short-acting gas and short-duration face-down positioning. Clin Ophthalmol.

[22] Wickens JC, Shah GK (2006). Outcomes of macular hole surgery and shortened face down positioning. Retina.

[23] Wong R, Gupta B, Williamson TH, Laidlaw DAH (2011). Day 1 postoperative intraocular pressure spike in vitreoretinal surgery (VDOP1). Acta Ophthalmol.

[24] Chen PP, Thompson JT (1997). Risk factors for elevated intraocular pressure after the use of intraocular gases in vitreoretinal surgery. Ophthalmic Surg Lasers.

[25] Sakamoto T, Hida T, Tano Y, Negi A, Takeuchi S, Ishibashi T, Committee on the use of long-lasting expanding gases in ophthalmology (2008). Survey of the use of long-lasting expanding gases in ophthalmology in Japan. Nihon Ganka Gakkai Zasshi.

[26] Heath G, Rahman R (2010). Combined 23-gauge, sutureless transconjunctival vitrectomy with phacoemulsification without face down posturing for the repair of idiopathic macular holes. Eye.

[27] Gosse E, Newsom R, Hall P, Lochhead J (2013). Changes in day 1 post-operative intraocular pressure following sutureless 23-gauge and conventional 20-gauge pars plana vitrectomy. Open Ophthalmol J.

[28] Ahn SJ, Woo SJ, Ahn J, Park KH (2012). Comparison of postoperative intraocular pressure changes between 23-gauge transconjunctival sutureless vitrectomy and conventional 20-gauge vitrectomy. Eye.

